# The Genetic Basis of Color Polymorphism in the Orb‐Web Spider *Gasteracantha cancriformis*

**DOI:** 10.1002/ece3.73315

**Published:** 2026-03-23

**Authors:** Paula Torres‐Quintero, Carolina Pardo‐Díaz, Fabian Salgado‐Roa, Camilo Salazar

**Affiliations:** ^1^ Department of Biology, School of Sciences and Engineering Universidad del Rosario Bogotá Colombia; ^2^ Department of Integrative Biology University of Texas at Austin Austin Texas USA

**Keywords:** color, *Gasteracantha cancriformis*, polymorphism, spider, transcriptomics

## Abstract

Animal coloration is a complex trait useful for studying adaptive evolution. In consequence, a major goal in evolutionary biology is to understand the genetic and developmental basis underlying coloration in animals and thus establish direct links between genetic and phenotype variability. Genomics tools have contributed the identification the specific genes and variants responsible for color variation in nonmodel animals, but the evidence is biased toward some taxa, while groups such as arachnids have been neglected. In this study, we aimed to identify genes that underlie coloration in spiders and generate genomic resources for these organisms. By deep sequencing RNA‐seq libraries from yellow, orange, white, and black female morphs of the color polymorphic spider *Gasteracantha cancriformis* , we assembled a transcriptome for this species and identified genes that were differentially expressed between color morphs. Among these, we detected genes with known roles in pigmentation pathways for carotenoids, melanin, ommochromes, and pteridines, suggesting that both pigmentary and structural coloration are involved in abdominal coloration in spiders. Signatures of positive selection on these genes suggest that abdominal coloration in *G. cancriformis* plays an adaptive role, although the specific selective pressures remain unknown. However, the fact that we identified more venom gland genes expressed in the most conspicuous morphs we tested (i.e., yellow and orange) suggests that abdominal coloration likely serves a defensive function against predators in these spiders. Overall, our results provide evidence that several pathways that control pigmentation in insects and vertebrates also play a role in arachnids.

## Graphical abstract

This study investigates the genetic basis of coloration in the polymorphic spider *Gasteracantha cancriformis* using RNA‐seq data from different female color morphs. We assembled a reference transcriptome and identified differentially expressed genes associated with known pigmentation pathways, including carotenoids, melanin, ommochromes, and pteridines. Evidence of positive selection and the expression of venom‐related genes in conspicuous morphs suggest that coloration in this species may have adaptive and defensive functions.

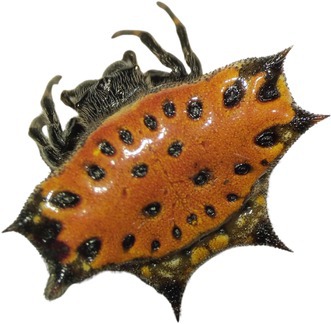

## 1 Introduction

Animal coloration is a complex and multifunctional trait, and its tractability has made it a powerful model system for studying adaptive evolution (Orteu and Jiggins 2020; San‐Jose and Roulin 2017; Vonholdt et al. 2021; Price‐Waldman and Stoddard 2021). Coloration affects various life history traits of an organism, such as courtship, camouflage/crypsis, defense/warning signaling, photoprotection, thermoregulation, and immunity (Vonholdt et al. 2021; Price‐Waldman and Stoddard 2021; Lu et al. 2024), thus playing a crucial role in animal fitness. In this context, understanding the genetic basis of coloration is a major goal in evolutionary biology that allows the establishment of a clear connection between genetic variants and phenotype while also providing information on the evolutionary forces driving biological diversity (Orteu and Jiggins 2020). In the last decade, genomics has fueled the identification of the precise genes and variants underlying color variation in non‐model animals, mostly vertebrates and some insects, especially Lepidoptera (San‐Jose and Roulin 2017; Price‐Waldman and Stoddard 2021; Lu et al. 2024).

Species exhibiting discrete phenotypic differences in coloration within their populations (i.e., color polymorphism) have emerged as an excellent model for identifying the genetic basis driving and maintaining intraspecific variation, especially that with adaptive value (Jamie and Meier 2020; Svensson 2017). In consequence, a growing catalog of color loci has begun to reveal the mutations, genes, and genetic architectures underlying color variation in the wild (Martin and Orgogozo 2013). The evidence shows that the most compelling examples of adaptive color polymorphisms are controlled by major effect loci (Orteu and Jiggins 2020) and are due to single mutations of large effect—SNPs (Garcia‐Elfring et al. 2023; Hoekstra et al. 2006), structural changes such as duplications or inversions (Brien et al. 2023; Jiggins 2016; Joron et al. 2011), changes in a master regulatory gene with multiple downstream targets (Nishikawa et al. 2015; Iijima et al. 2018), or changes in regulatory regions (Orteu, Hornett, et al. 2024; Orteu, Kucka, et al. 2024). However, this understanding mostly comes from a limited subset of taxa, including butterflies, wood tiger moths, rodents, birds, frogs, and fishes, and usually from the implementation of a candidate gene approach (Orteu and Jiggins 2020).

Research on spider coloration has largely focused on elucidating the pigmentary and mechanistic bases of coloration, as well as its functional significance (Gawryszewski et al. 2015; Hsiung et al. 2019; Salgado‐Roa et al. 2023; Gawryszewski and Motta 2012). However, the identification of the genetic basis of coloration is still in its infancy, and few studies have characterized the pathways or candidate genes underlying this phenotype (Hsiung et al. 2019; Croucher et al. 2013; Yim et al. 2014; Salgado‐Roa et al. 2022). Thus, to date, we know that body coloration in spiders is the result of the presence of pigments such as melanin that control black and brown (Hsiung et al. 2015), carotenoids that produce yellow (Hsiung et al. 2017), ommochromes that produce a variety of colors including yellow, orange, red, brown, and black (Hsiung et al. 2017), and bilins that are mostly green (Holl and Rüdiger 1975). Similarly, microstructures contribute to spider coloration via the presence, shape, and arrangement of guanine crystals, resulting in different visual appearances, including white, silver and yellow (Gawryszewski et al. 2015; Holl and Rüdiger 1975), or even bright and metallic blue in the case of guanine crystal‐based iridophores (Hsiung et al. 2019). Also, guanine crystals are involved in UV reflection that mediates not only color perception but also color variation within days (Gawryszewski et al. 2015). Despite this knowledge, spiders still lack a detailed understanding of the genetic basis of pigmentary and structural coloration production.

Arachnids are among the most diverse and abundant groups of animals, with over 50,000 described species (Heads and Grehan 2021; Buchholz et al. 2020; World Spider Catalog 2026), of which, several display spectacular color polymorphisms (Croucher et al. 2013; Oxford 2005; Oxford and Gillespie 1998; Salgado‐Roa, Stuart‐Fox, White, and Medina 2024). In particular, *Gasteracantha cancriformis* is a color polymorphic orb‐web spider in which females show a striking variation in the coloration of the abdomen both across the distribution of the species, but also within the same population (Salgado‐Roa et al. 2022, 2018; World Spider Catalog 2026). Males, however, are monomorphic and black (World Spider Catalog 2026; Muma 1971), although the causes of this variation remain unknown (Salgado‐Roa et al. 2023; Gawryszewski and Motta 2012). Such polymorphism combined with a growing list of genetic resources for the species make it a promising system for investigating the genetic basis of color production and variation in spiders. In this study, we explored the genetic basis of female color polymorphism in *G. cancriformis* by implementing a transcriptomics analysis of multiple color morphs from a single locality (i.e., black, yellow, orange, and white). Following this approach, we expect to find: (i) melanin related genes highly expressed in the black morph, (ii) carotenoid and ommochrome genes overexpressed in the yellow and orange morphs, and (iii) underexpression of pigment related genes in the white morph coupled with higher expression of genes associated with guanine crystals. To the best of our knowledge, this is one of the few studies that addresses this question in color polymorphic spiders, a group of animals not included in the most up to date catalog of color loci (Martin and Orgogozo 2013).

## 2 Materials and Methods

### 2.1 Sampling, RNA Extraction, and Sequencing

We collected 29 adult females of *G. cancriformis* in Ibagué, Colombia (4° 37′ N, −75° 15′ W), that included four color morphs (Table S1): (i) yellow; *n* = 8, (ii) black; *n* = 5, (iii) white; *n* = 8, (iv) orange; *n* = 8. Individuals were immediately preserved in Trizol and stored at −80°C. We extracted RNA using the RNeasy Mini Kit (Qiagen). First, we fragmented the samples (whole body) with a TissueLyser III (Qiagen) and left the samples in Trizol at 4°C for 7 days. Then, we followed the standard Trizol‐chloroform extraction method as specified by Qiagen. RNA concentration and quality were assessed by agarose gel, Qubit and Bioanalyzer. Paired‐end sequencing (150 bp) was performed on an Illumina HiSeq 4000 by Novogen.

### 2.2 De Novo Transcriptome Assembly

Fastq files were inspected for quality with FastQC v.0.11.9 (Andrews 2010), erroneous *k*‐mers were removed with rCorrector v1.0.5 (Song and Florea 2015), and sequences with Phred score < 30 were removed with Trim Galore v0.6.7 (Krueger 2017). We used Trinity v2.14.0 (Grabherr et al. 2011) with default parameters, except for the minimum contig length that was set to 2000 bp, to generate a *de novo* transcriptome assembly for *G. cancriformis* . To reduce the complexity of the assembly and identify true transcripts and isoforms, we removed duplicated and misassembled sequences by clustering similar transcripts into groups using CDHIT‐est v4.8.1 (Li and Godzik 2006). Then, assembly completeness was assessed using the Araneae data set (araneae_odb12) of BUSCO v5.4.0 (Simão et al. 2015). The assembly was then repeat‐masked using a combination of the de novo repeat finder RepeatModeler v2.0.1 (Smit and Hubley 2008) and the homology‐based repeat finder RepeatMasker v4.1.5 (https://repeatmasker.org/). Finally, assembly statistics were calculated by running the perl script TrinityStats.pl. from Trinity v2.14.0 (Grabherr et al. 2011). The resulting transcriptome was aligned against the existing transcriptome of two species of *Macracantha* ( *M. arcuata* and *M. hasselti* ) to compare our assembly to those already available in the genus. For this, we used BLAST v3.2.1 (Kent 2002) and downloaded the sequences from the NCBI Bioproject PRJNA219237 (Zhao et al. 2014).

We applied the same procedure to generate four additional color specific assemblies using reads from individuals from the same morph. These were used in the analysis for signatures of selection (see below).

### 2.3 Functional Annotation

All transcripts in the final assembly were identified by blasting them against the NCBI's non‐redundant (nr) database (accessed October 22, 2023) using BLASTx Diamond v2.1.8 (Buchfink et al. 2021). We used the best local alignment at an e‐value cutoff of 1e‐5 and retained up to 10 best hits per transcript. Then, transcripts were functionally annotated using Blast2GO v6.0 (Conesa et al. 2005) to predict Gene Ontology (GO) terms and to analyze protein domains with the InterProScan (Jones et al. 2014). We then filtered out transcripts with Blastx_Hit_Taxonomy_Name matching virus or bacteria to remove any potential contamination. The assembled sequences were also assigned to the Kyoto Encyclopedia of Genes and Genomes (KEGG) pathways using the KEGG Automatic Annotation Server (KAAS) (Kanehisa et al. 2016) with the bi‐directional best hit (BBH) alignment method and the gene dataset manually curated to include 35 arthropods (including insects, crustaceans, and chelicerates; Table S2). Next, the expressed transcripts were translated into potential proteins according to ORF prediction by TransDecoder v.5.7.1 (Haas et al. 2013), and the potential coding transcripts were also annotated against the Swiss‐Prot database (*e*‐value of 1e‐5).

### 2.4 Read Mapping and Differential Expression Analyses

The reference transcriptome index was constructed using Bowtie2 (Langmead and Salzberg 2012) using the draft transcriptome constructed above. We then aligned the clean paired‐end reads of each sample to the reference transcriptome, and estimated transcript abundance per individual using the Trinity script *align_and_estimate_abundance.pl* (http://trinityrnaseq.github.io/) with ‐‐*est_method RSEM ‐‐aln_method bowtie2*. Read counts from all samples were combined into a single matrix using the *abundance_estimate_to_matrix.pl* script implemented in Trinity v2.14.0 (Grabherr et al. 2011). We used DESeq2 v3.19 (Love et al. 2014) to normalize expression estimates with counts scaled by the total number of reads (CPM—counts per million) and conduct the differential gene expression (DGE) analysis. Six DGE comparisons between color morphs were performed: (i) yellow vs. orange, (ii) yellow vs. white, (iii) yellow vs. black, (iv) orange vs. white, (v) orange vs. black, and (vi) white vs. black. Differentially expressed transcripts were identified using a likelihood ratio test with the glmLRT function, and *p* values were adjusted using the Benjamini–Hochberg false discovery rate (FDR). Only genes with FDR < 0.05 were considered as differentially expressed. Then *GOseq* package in R (Young et al. 2010) conduct a GO enrichment analysis with the differentially expressed genes.

### 2.5 Detection of Pigment and Venom Pathway Genes

We identified 568 transcripts that were differentially expressed in a color associated pattern. We analyzed these transcripts for associations with pigment synthesis using them as query in two BLASTx searches conducted with Diamond v2.1.8 (Buchfink et al. 2021). In the first search, we used the protein sequences from the AmiGO category “metabolic process of pigment” in *Drosophila melanogaster* as database (Carbon et al. 2009). Transcripts with significant hits (*e*‐value of 1e‐3) were further used in a reciprocal BLASTx search against the *D. melanogaster* reference proteome from UniProt knowledgebase (Celniker et al. 2002). In the second search, we used all orthologous for the 41 genes comprised in the GO terms: *GO:0048067 cuticle pigmentation, GO:0043474 pigment metabolic process involved in pigmentation, GO:0043473 pigmentation, GO:0090740 integral component of pigment granule membrane, GO:0048753 pigment granule organization, GO:0006727 ommochrome biosynthetic process, GO:0006582 melanin metabolic process, GO:0042438 melanin biosynthetic process, and GO:0016063 rhodopsin biosynthetic process* in *D. melanogaster* as database. These were identified by following the links for each of these genes available at https://www.geneweaver.org.

We also analyzed the 568 transcripts for associations with venom/toxin genes. For this, we used them as query in a BLASTx search against a database of 7680 protein sequences with known associations with venoms and toxins. These were identified and retrieved from arachnoserver (https://arachnoserver.qfab.org/mainMenu.html), NCBI (https://www.ncbi.nlm.nih.gov/), and venomzone (https://venomzone.expasy.org/). Transcripts with significant hits (*e*‐value < 1e‐5 and identity > 90%) were considered as venom or toxin related.

We then assessed differences in expression between color morphs in pigment pathways or venom/toxin genes by applying a Kruskal–Wallis post hoc Dunn test with Holm's correction; statistical significance defined as *p* < 0.05. This was done with the R package ggstatsplot (Patil 2021).

### 2.6 Signatures of Selection

We created a fasta file for each of the 568 differentially expressed transcripts identified above. Each file contains the sequences for the same transcript that were retrieved from each color specific assembly (when present). These were processed in Translatorx v14.0 (Abascal et al. 2010) to align and translate each transcript using the MAFFT algorithm (Katoh and Standley 2013). We visually inspected these files for stop codons and misalignments, and only those with few gaps were retained. Next, we used PAL2NAL v14.1 (Suyama et al. 2006) to construct a codon alignment per transcript, and each codon alignment was converted to Phylip format with FASconCAT‐G v1.05.1 (Kück and Longo 2014).

Each alignment was used as input to construct a maximum likelihood tree using RAxML v8.2.11 (Stamatakis 2014) with the GTRGAMMA model. Node support was estimated using 1000 ultrafast bootstrap replicates. The resulting tree as well as the Phylip alignment per transcript were used as input for CODEML (Álvarez‐Carretero et al. 2023) in the PAMLv4.10.6 package (Yang 2007) to detect signatures of positive selection and evolutionary constraints. For the latter, we followed the protocol of (Álvarez‐Carretero et al. 2023) and estimated pairwise rates of nonsynonymous (dN) and synonymous (dS) substitutions between color morphs for each of the 61 genes found to be associated with color pattern under two models, one of neutrality (fixed omega = 0) and one of positive selection (fixed omega = 1). Then, we assessed the statistical significance of these results carrying out a likelihood ratio test (LRT) analysis with one degree of freedom. A *p* < 0.01 was considered significant and, thus, indicative of positive selection.

## 3 Results

### 3.1 De Novo Transcriptome Assembly

A total of 1,373,443,238 reads were generated across all 29 individuals with a mean quality score of 36 and an average of 47,360,111 reads per individual (Table S1). After quality filtering, we kept 1,345,208,518 reads with an average of 46,386,501 reads per individual (Table S1). The TRINITY‐based assembly for *G. cancriformis* obtained by pooling the reads from all individuals had a GC content of 32.66% and returned 23,587 transcripts that were clustered into 14,625 genes (Table 1). These transcripts had a maximum length of 24,924 bp, mean length of 2987 bp, minimum length of 1980 bp, and the N50 was 3685 bp (Table 1 and Figure S1). Over 80% of the transcripts in *M. arcuata* and *M. hasselti* were found in our reference assembly (identity > 75% and length > 200 bp), but our assembly had a higher N50 and fewer genes and transcripts (Table 1).

**TABLE 1:** Summary statistics for the assembly of *G. cancriformis* compared to those of *G. arcuata* and *G. hasselti* .

	*G. cancriformis*	*G. arcuata*	*G. hasselti*
Total length (bp)	84,474,406	23,437,402	34,353,081
N50 (bp)	3685	1075	1231
Number of unigenes	14,625	54,875	75,455
Number of transcripts	23,587	—	—
Mean contig length (bp)	2987	—	—
Minimum contig length (bp)	1980	—	—
Maximum contig length (bp)	24,924	—	—

The quality and completeness analysis of BUSCO indicates that the assembly of *G. cancriformis* contains 63.4% of the expected gene content in arachnids, of which 60.8% are single copy genes. BUSCO also revealed that repetitive regions accounted for 39.1% of the *G. cancriformis* assembly.

### 3.2 Functional Annotation

Annotation of the final assembly resulted in 20,456 annotated transcripts, of which 3312 were filtered out due to Blastx_Hit_Taxonomy_Name match with virus or bacteria, leaving 17,144 annotated transcripts (Table S3). Of those, 16,964 (98.95%) found a match with BLASTx in the NR database, and 13,206 (77.02%) transcripts found a match with BLASTp in the Swiss‐Prot database. The KEGG annotation assigned 8988 (38.10%) transcripts to 4276 KEGG terms divided into five categories (Figure S2A). Finally, 8779 transcripts (37.21%) were assigned to 2163 GO terms, whereas more than 60% were of unknown function. The major GO categories were biological process (80.49%), cellular component (38.47%), and molecular function (62.7%; Figure S2B).

### 3.3 Differential Expression Analyses

**FIGURE 1:**
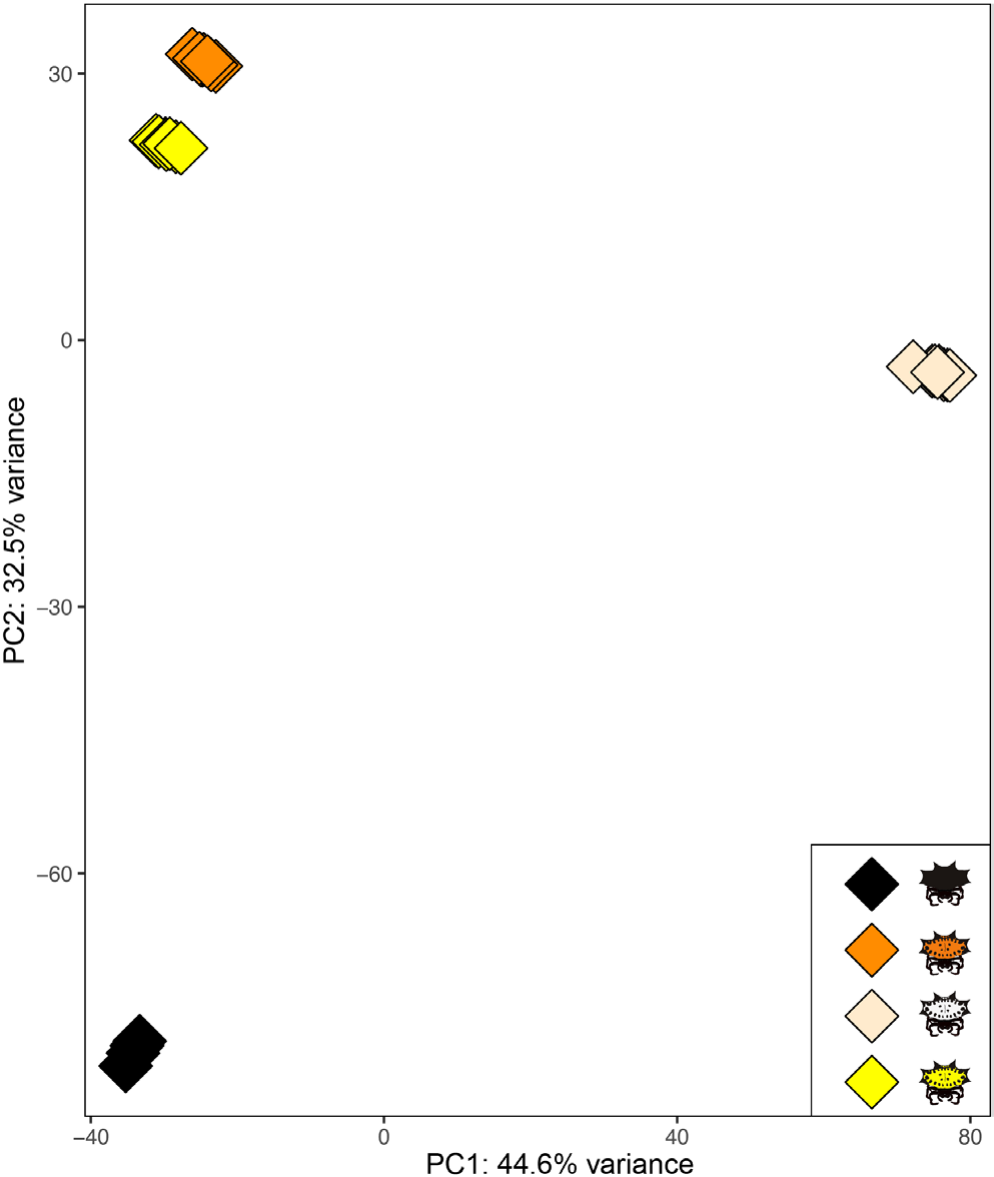
Principal component analysis (PCA) plot for color associated DETs shows clustering by color morph. Each symbol represents an individual sample.

**FIGURE 2:**
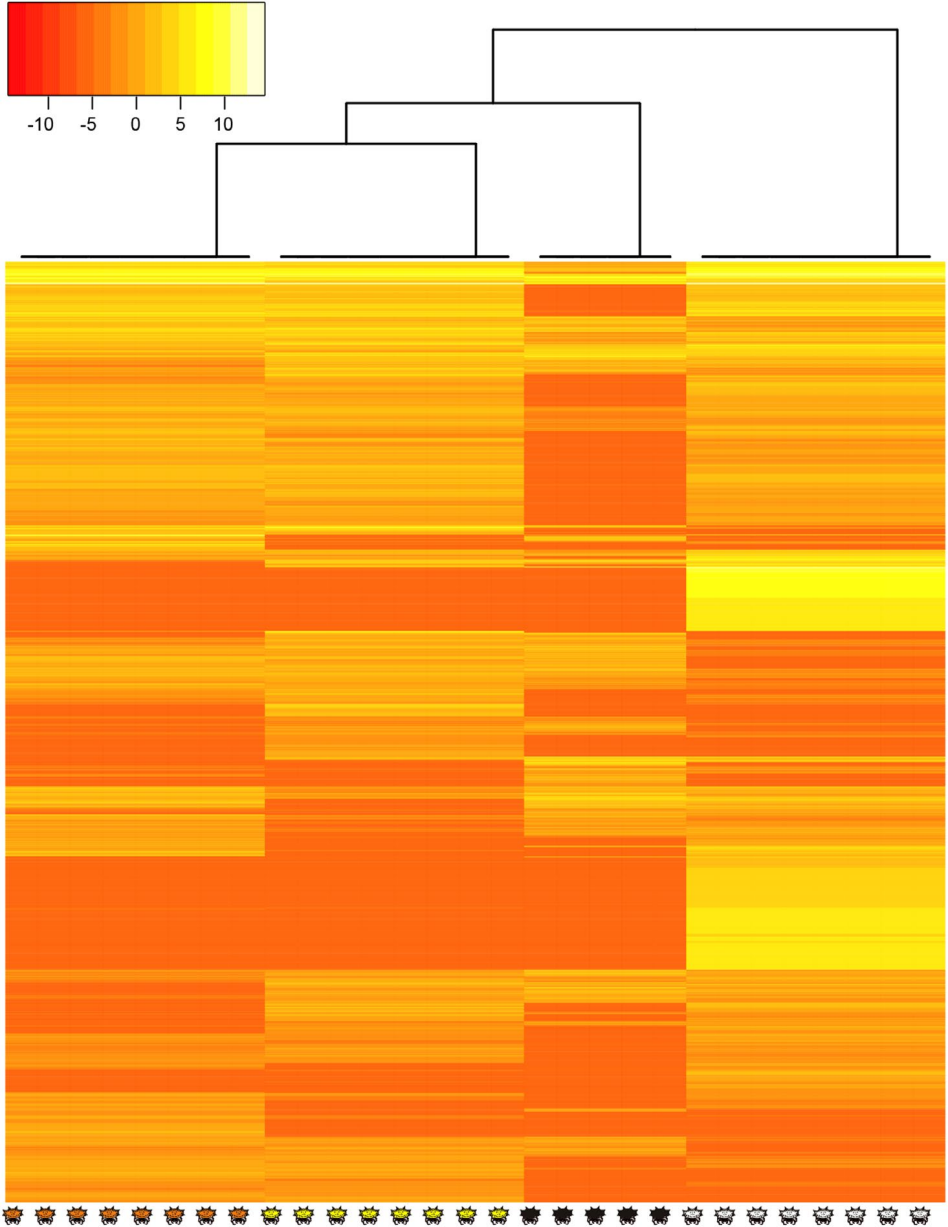
Cluster analysis of differential transcript expression. Heatmap of the top 100 more variable DETs between black, yellow, orange, and white morphs. Expression values for each transcript are normalized across all samples (columns) by the Z‐score where red and yellow mean the lowest and higher expression values, respectively.

We identified 2029 transcripts (1761 unigenes) that were differentially expressed between color morphs at log_2_FC ≥ 1.3 in at least one comparison (FDR ≤ 0.05; Table S4); 568 of these transcripts were differentially expressed in a color associated pattern (Table S5). The PCA of all 2029 DETs revealed that 77.1% of the variation in expression in these transcripts is explained by the first two principal components (Figure 1). The first component explains 44.6% of the variation in expression and clearly separates white individuals from any other morph, while the second component explains 32.5% of the variation and separates pigmented individuals based on their color morph. In this way, we observed three main groups: (i) black, (ii) orange, and (iii) yellow, with the latter two (i.e., spectral morphs) being closer. This pattern is entirely consistent with the heatmap (Figure 2), where white individuals form a well separated cluster. All other morphs form a big cluster where the yellow and orange morphs group together and are separated from the black individuals.

**FIGURE 3:**
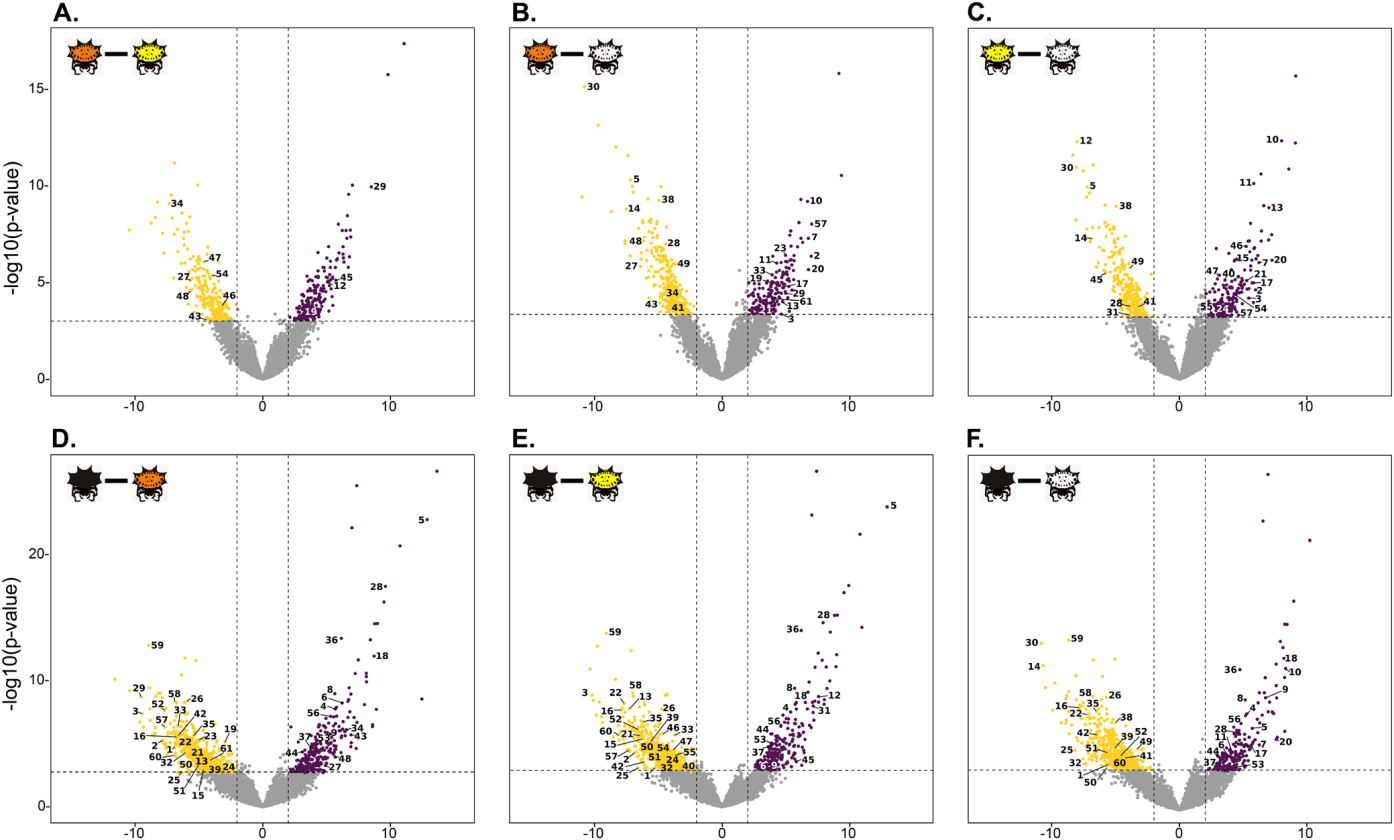
Volcano plots of DETs in each comparison. (A) Orange vs. Yellow; (B) Orange vs. White; (C) Yellow vs. White; (D) Black vs. Orange; (E) Black vs. Yellow; (F) Black vs. White. Purple dots indicate upregulated, yellow indicate downregulated, and gray indicate not significant. Numbers indicate DETs associated with pigmentation and detailed in Table 3.

The comparison with the least differential expression was between spectral colored morphs (yellow and orange; 437 transcripts), where 51.3% of the differentially expressed transcripts (DETs) were upregulated and 48.7% were downregulated in the orange morph (Table 2, Figure 3A and Table S4). In contrast, we found the highest differential expression when comparing the black morph versus any of the spectral colored morphs (yellow or orange; Tables 2, S4 and Figure 3D,E). In both comparisons ~70% of the DETs were downregulated in the black morph. Comparisons involving the white morph showed intermediate DET values (Table 2, Figure 3C,F and Table S4).

**TABLE 2:** Summary of differential expression between color morphs.

Comparison		DETs	Upregulated in the first morph	Downregulated in the first morph
Orange—Yellow	OY	437	224	213
Orange—White	OW	458	198	260
Yellow—White	YW	452	194	258
Black—Orange	BO	919	288	631
Black—Yellow	BY	781	238	543
Black—White	BW	722	192	530

### 3.4 Detection of Pigment Pathway Genes

In our set of DETs, we identified 61 transcripts associated with 56 pigment genes (Table 3). Among these, five were associated with the presence of any pigment (black, orange, or yellow), being consistently overexpressed in pigmented morphs when compared against the white form (Table 3 and Figure 3B,C,F). These transcripts have known functions in the ommochromes or pteridine pathways. Other eight DETs were associated with having colored abdomen and thus were always overexpressed in the yellow and orange morphs versus their white or black counterparts (Table 3 and Figure 3B–E). These transcripts have known roles in the synthesis of carotenoids, ommochromes, and pteridine, and also in the inhibition of melanin. We also found two transcripts with expression consistently associated with the absence of yellow or orange, as they were always underexpressed in these phenotypes compared with the black or white phenotypes (Table 3 and Figure 3B–E). Both transcripts have known roles in melanism. Interestingly, most of the DETs identified were associated with the presence or absence of the black phenotype. For instance, 10 DETs had expression perfectly matching the presence of black and were always overexpressed in the black morph in all the comparisons tested (Table 3 and Figure 3D–F). Consistently, most of these genes play roles in the production of melanin. In contrast, 15 DETs had the exact opposite expression pattern that perfectly matches the absence of black and were always underexpressed in the black morph in all the comparisons tested (Table 3 and Figure 3D–F). These have reported functions in the inhibition of melanin or synthesis of rhodopsins. Similarly, we found DETs associated with the presence/absence of yellow, with five DETs always overexpressed and two DETs consistently underexpressed in the yellow morph in all comparisons involving this phenotype (Table 3 and Figure 3A,C,E). Additionally, we found four DETs that were always overexpressed and four DETs that were always underexpressed in the orange morph, thus being associated with the presence/absence of this phenotype (Table 3 and Figure 3A,B,D). Finally, we identified five DETs always upregulated in the white morph and thus associated with this phenotype (Table 3 and Figure 3B,C,F) and were the only set of genes with any sort of function in the synthesis of chitin and iridophore.

**TABLE 3:** DETs associated with color phenotype and known role in pigmentation. Comparisons are denoted as follows: BO (black vs. orange), BY (black vs. yellow), BW (black vs. white), OW (orange vs. white), YW (yellow vs. white), and OY (orange vs. yellow). Differential expression in each gen and comparison is indicated by DE, and the phenotype where the gene is overexpressed is colored coded in accordance with phenotype. Code refers to numbers in Figure 3, with asterisk (*) indicating those genes with signatures of positive selection.

Trans ID	Gene ID	Gene name	Gene description	Pattern of differential expression						Role known in	Code	Duplicated
				BO	BY	BW	OW	YW	OY			
*Presence of pigment (yellow/orange/black)*												
TRINITY_DN20798_c0_g1_i1	XM_022062280.1	loco	regulator of G‐protein signaling loco	—	—	DE	DE	DE	—	Ommochrome	11	No
TRINITY_DN6340_c0_g1_i5	XM_019917639.2	w	white	—	—	DE	DE	DE	—	Ommochrome	7	Yes
TRINITY_DN8102_c0_g1_i4	XM_045642044.1	Hayan	serine protease Hayan			DE	DE	DE	—	Ommochrome and Pteridine	10	No
TRINITY_DN1418_c0_g1_i4	NM_079077.4	Pu	GTP cyclohydrolase punch	—	—	DE	DE	DE	—	Pteridine	20	No
TRINITY_DN12730_c0_g1_i3	GBN00013.1	NFI	Nuclear factor 1	—	—	DE	DE	DE	—	Pteridine	17	Yes
*Presence of color (yellow/orange)*												
TRINITY_DN13732_c0_g1_i5	XM_023180971.2	DCX‐EMAP	Doublecortin‐domain‐containing echinoderm‐microtubule‐associated protein	DE	DE	—	DE	—	—	NA	33	No
TRINITY_DN3892_c0_g1_i18	XM_004922787.5	p	pink	DE	DE	—	DE	DE	—	Ommochrome and Pteridine	57	No
TRINITY_DN316_c1_g2_i1	PIO56948.1	astacin	Astacin	DE	DE	—	DE	DE	—	Carotenoid	2	No
TRINITY_DN7747_c0_g1_i3	GBM04805.1	Vg	Vitellogenin	DE	DE	—	DE	DE	—	Carotenoid	3	No
TRINITY_DN7271_c0_g1_i2	GBM77979.1	CYP	Cytochrome P450 18a1	DE	DE	—	DE	DE	—	Carotenoid	13*	Yes
TRINITY_DN786_c0_g1_i3	NM_079077.4	Pu	GTP cyclohydrolase punch	DE	DE	—	DE	DE	—	Pteridine	21	No
TRINITY_DN32763_c0_g1_i1	XM_058271116.1	HoxA5	homeobox protein Hox‐A5	DE	DE	—	—	DE	—	NA	24	No
TRINITY_DN4650_c0_g1_i25	XM_011138866.3	cn	Kynurenine 3‐monooxygenase (cinnabar)	DE	DE	—	—	DE	—	Ommochrome	15	No
*Absence of color (yellow/orange)*												
TRINITY_DN4886_c0_g1_i1	NM_141193.4	MP1	Melanization protease 1	DE	DE	DE	DE	DE	—	Melanin	5	No
TRINITY_DN3894_c0_g1_i3	XM_030513398.1	MP2	Melanization Protease 2	DE	DE	DE	DE	DE	—	Melanin	28	No
*Presence of black*												
TRINITY_DN20333_c1_g2_i1	XM_043215117.1	Abd‐B	homeobox protein abdominal‐B	DE	DE	DE	—	—	—	Melanin	56	No
TRINITY_DN31201_c0_g1_i4	XM_035880733.1	Cdk5alpha	Cdk5 activator‐like protein	DE	DE	DE	—	—	—	Melanin	18	Yes
TRINITY_DN10288_c0_g1_i5	XM_025088066.1	ATP7	copper‐transporting ATPase 7	DE	DE	DE	—	—	—	Melanin	8	No
TRINITY_DN1640_c0_g1_i2	XM_004533387.2	PPO1	Prophenoloxidase 1	DE	DE	DE	—	—	—	Melanin	53*	No
TRINITY_DN2409_c0_g1_i6	NM_134309.2	Rho1	Rho1	DE	DE	DE	—	—	—	Melanin	36	Yes
TRINITY_DN4263_c0_g1_i1	NM_001299308.1	lightoid	Rab32/RP1 (lightoid)	DE	DE	DE	—	—	—	Melanin	6	No
TRINITY_DN6898_c0_g1_i1	NM_001272712.1	B‐H2	BarH2	DE	DE	DE	—	—	—	Melanin	9*	Yes
TRINITY_DN17285_c0_g1_i6	XM_046541774.1	RhoGEF3	Rho guanine nucleotide exchange factor 3	DE	DE	DE	—	—	—	Melanin	37	No
TRINITY_DN2458_c0_g1_i35	XM_050641341.1	cd	cardinal	DE	DE	DE	—	—	—	Ommochrome	4	No
TRINITY_DN8164_c0_g1_i1	XM_053835225.1	Burs	Cuticle‐tanning hormone *bursicon*	DE	DE	DE	—	—	—	Melanin	44	No
*Absence of black*												
TRINITY_DN13162_c0_g1_i13	XM_044899347.1	e	ebony	DE	DE	DE	—	—	—	Melanin (inhibition)	59	No
TRINITY_DN5395_c0_g1_i8	XM_006613942.2	S6kII	Ribosomal protein S6 kinase II	DE	DE	DE	—	—	—	Melanin (inhibition)	51	No
TRINITY_DN35702_c0_g1_i2	XM_035876088.1	kar	monocarboxylate transporter 10‐like protein kar	DE	DE	DE	—	—	—	Ommochrome	39	No
TRINITY_DN4719_c0_g1_i2	XM_063376638.1	ninaG	neither inactivation nor afterpotential protein G	DE	DE	DE	—	—	—	Rhodopsin	58	No
TRINITY_DN13732_c0_g1_i8	XM_050451792.1	Glut1	Glucose transporter 1	DE	DE	DE	—	—	—	Melanin (inhibition)	60	No
TRINITY_DN3144_c1_g1_i4	XM_050451792.1	Glut1	Glucose transporter 1	DE	DE	DE	—	—	—	Melanin (inhibition)	42*	No
TRINITY_DN11748_c0_g1_i15	XM_011299316.1	Glut1	Glucose transporter 1	DE	DE	DE	—	—	—	Melanin (inhibition)	32	No
TRINITY_DN6380_c0_g1_i1	NM_001043301.2	Cnx99A	calnexin 99	DE	DE	DE	—	—	—	Rhodopsin	22	No
TRINITY_DN669_c0_g3_i4	XM_058949491.1	bab1	bric a brac	DE	DE	DE	—	—	—	Melanin (inhibition)	35	No
TRINITY_DN17459_c0_g1_i1	XM_055998953.1	LOC129918432	leucine‐rich repeat‐containing protein 4C	DE	DE	DE	—	—	—	NA	25	No
TRINITY_DN19911_c0_g1_i4	XM_053738640.1	btn	buttonless	DE	DE	DE	—	—	—	NA	26	No
TRINITY_DN3631_c0_g1_i2	NM_057308.4	ninaA	neither inactivation nor after potentialA	DE	DE	DE	—	—	—	Rhodopsin	16	Yes
TRINITY_DN28029_c0_g1_i2	XM_065370320.1	Klp61F	Kinesin‐like protein at 61F	DE	DE	DE	—	—	—	NA	50	Yes
TRINITY_DN935_c0_g1_i27	XM_031499455.1	Klp61F	Kinesin‐like protein at 61F	DE	DE	DE	—	—	—	NA	52	No
TRINITY_DN1048_c1_g1_i4	XM_065478058.1	Shc	SHC‐adaptor protein (tyrosine kinase substrates)	DE	DE	DE	—	—	—	NA	1	Yes
*Presence of yellow*												
TRINITY_DN16106_c0_g1_i2	XM_048660797.1	S6kII	Ribosomal protein S6 kinase II (receptor tyrosine kinase)	—	DE	—	—	DE	—	Melanin (inhibition)	40	No
TRINITY_DN20172_c0_g1_i5	XM_047489343.1	Zir	Zizimin‐related	—	DE	—	—	DE	—	Melanin	55	No
TRINITY_DN5661_c0_g1_i2	XM_065508605.1	Hayan	serine protease Hayan	DE	DE	—	—	DE	DE	Ommochrome and Pteridine	47	No
TRINITY_DN5661_c0_g1_i19	XM_037868574.1	Hayan	serine protease Hayan	—	DE	—	—	DE	DE	Ommochrome and Pteridine	46	No
TRINITY_DN3240_c0_g1_i9	XM_023064617.1	Glut1	Glucose transporter 1	—	DE	—	—	DE	DE	Melanin (inhibition)	54	Yes
TRINITY_DN5032_c0_g1_i3	XM_038266828.1	Eph	Eph receptor tyrosine kinase	—	DE	—	—	DE	DE	Melanin	45	Yes
*Absence of yellow*												
TRINITY_DN12636_c0_g1_i19	XM_028287758.2	pns	pinstripe	—	DE	—	—	DE	DE	Melanin	31	No
TRINITY_DN7536_c0_g1_i11	XM_042289430.1	EphA5	Ephrin‐A5	—	DE	—	—	DE	DE	Heme	12	Yes
*Presence of orange*												
TRINITY_DN843_c0_g1_i2	XM_055462457.1	Eph	Eph receptor tyrosine kinase	DE	—	—	DE	—	DE	Melanin	29	No
TRINITY_DN27843_c0_g1_i1	XM_038044568.1	Vav	Vav guanine nucleotide exchange factor	DE	—	—	DE	—	DE	Melanin	19	No
TRINITY_DN7747_c0_g1_i6	GBM04805.1	Vg	Vitellogenin	DE	—	—	DE	—	—	Carotenoid	23	No
TRINITY_DN27887_c0_g1_i6	XM_050451792.1	Glut1	Glucose transporter 1	DE	—	—	DE	—	—	Rhodopsin	61	No
*Absence of orange*												
TRINITY_DN5173_c0_g1_i6	XM_021839075.1	Eph	Eph receptor tyrosine kinase	DE	—	—	DE	—	DE	Melanin	43	No
TRINITY_DN2713_c0_g1_i21	XM_014386959.2	Vav	Vav guanine nucleotide exchange factor	DE	—	—	DE	—	DE	Melanin	34	Yes
TRINITY_DN4771_c0_g1_i8	XM_031990077.1	Ire1	serine/threonine‐protein kinase/endoribonuclease Ire1	DE	—	—	DE	—	DE	NA	48*	No
TRINITY_DN4813_c0_g1_i3	XM_053738640.1	btn	buttonless	DE	—	—	DE	—	DE	NA	27	No
*Presence of white*												
TRINITY_DN6824_c0_g1_i3	XM_053842569.1	rdhB	retinol dehydrogenase B	—	—	DE	DE	DE	—	Rhodopsin	38	No
TRINITY_DN2625_c0_g1_i12	XM_055686153.1	chs‐2	Chitin synthase chs‐2	—	—	DE	DE	DE	—	Chitin	30	No
TRINITY_DN2065_c0_g1_i2	GBN14364.1	karneol	Endothelin‐converting enzyme 2 ECE‐2	—	—	DE	DE	DE	—	Iridophore	14	No
TRINITY_DN16939_c0_g1_i12	XM_018198010.1	Exn	Ephexin	—	—	DE	DE	DE	—	Melanin (inhibition)	41	No
TRINITY_DN17435_c0_g1_i1	XM_042291061.1	Eph	Eph receptor tyrosine kinase	—	—	DE	DE	DE	—	Melanin	49	No

**FIGURE 4:**
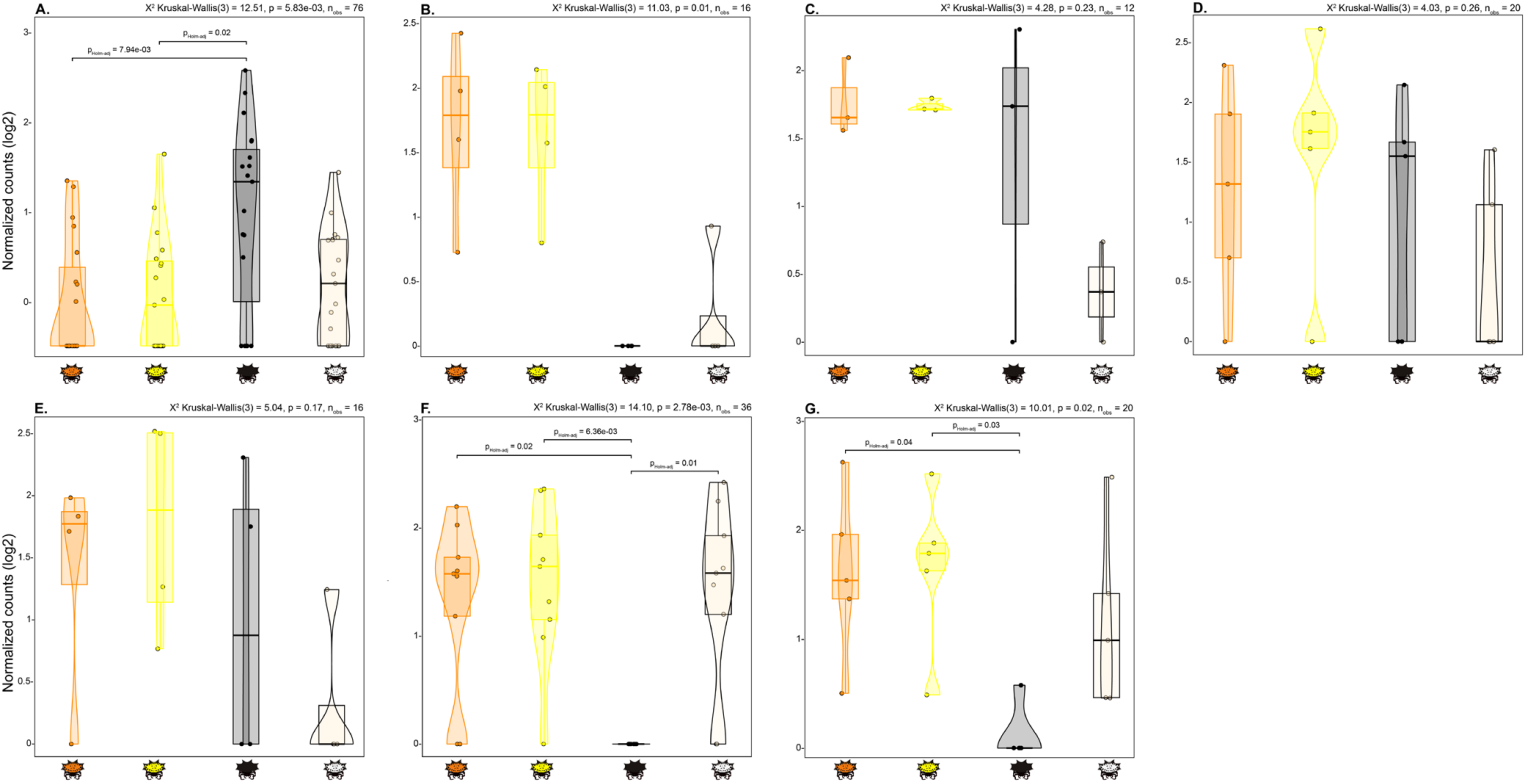
Comparison of expression of genes involved in pigmentation among color morphs. (A) Melanin, (B) Carotenoid, (C) Pteridine, (D) Ommochrome, (E) Ommochrome + Pteridine, (F) Rhodopsin., and (G) Melanin (inhibition). Only DETs included in Table 3 were used in this comparison.

Overall, the black morph is characterized as having the highest expression of melanin related genes (Figure 4A) and the lowest expression of rhodopsin and melanin inhibition genes (Figure 4F,G). The white morph had the lowest expression of pteridine genes (Figure 4C) and low expression of carotenoid genes (Figure 4B), but a high expression of rhodopsin genes (Figure 4F). The yellow and orange morphs have a similar expression pattern characterized by the highest expression of carotenoids and melanin inhibition genes (Figure 4B,G), and a lower expression of melanin genes (Figure 4A).

Interestingly, 10 of the DETs between color morphs had been documented as transcripts present in venom glands in animals (Table 4). Of these, six are overexpressed in the orange or yellow morphs, four in the black morph, and none in the white morph.

**TABLE 4:** DETs associated with color phenotype and known presence in venom glands. Comparisons are denoted as follows: BO (black vs. orange), BY (black vs. yellow), BW (black vs. white), OW (orange vs. white), YW (yellow vs. white), and OY (orange vs. yellow). Differential expression in each gen and comparison is indicated by DE, and the phenotype where the gene is overexpressed is colored coded in accordance with phenotype.

Trans ID	Gene ID	Gene description	Pattern of differential expression						Duplicated
			BO	BY	BW	OW	YW	OY	
TRINITY_DN2993_c1_g1_i2	ABX75386	60S ribosomal protein L40A	DE	—	—	DE	—	DE	No
TRINITY_DN768_c0_g1_i19	AII97912	BLTX547	DE	—	—	DE	—	DE	Yes
TRINITY_DN3668_c0_g1_i9	GFT00695	snake venom metalloproteinase‐disintegrin‐like mocarhagin	DE	—	DE	—	—	—	No
TRINITY_DN24411_c0_g1_i4	PRD33046	Snake venom metalloproteinase acutolysin‐C	DE	DE	—	DE	DE	—	No
TRINITY_DN2706_c6_g1_i3	KAF8773531.1	Disintegrin and metalloproteinase like protein	—	—	—	DE	DE	—	No
TRINITY_DN1144_c0_g1_i14	AII97892	BLTX527	—	DE	—	—	DE	DE	No
TRINITY_DN1640_c0_g1_i2	QHA25218	hemocyanin subunit A, partial	DE	DE	DE	—	—	—	No
TRINITY_DN3167_c0_g1_i4	QNF22887	putative heat shock protein isoform 2	DE	DE	DE	—	—	—	No
TRINITY_DN1319_c0_g1_i8	GIY31601.1	Spermine synthase	DE	DE	DE	—	—	—	Yes
TRINITY_DN8026_c0_g1_i11	ABA62021	Dermonecrotic toxin isoform 1	DE	DE	DE				No

**FIGURE 5:**
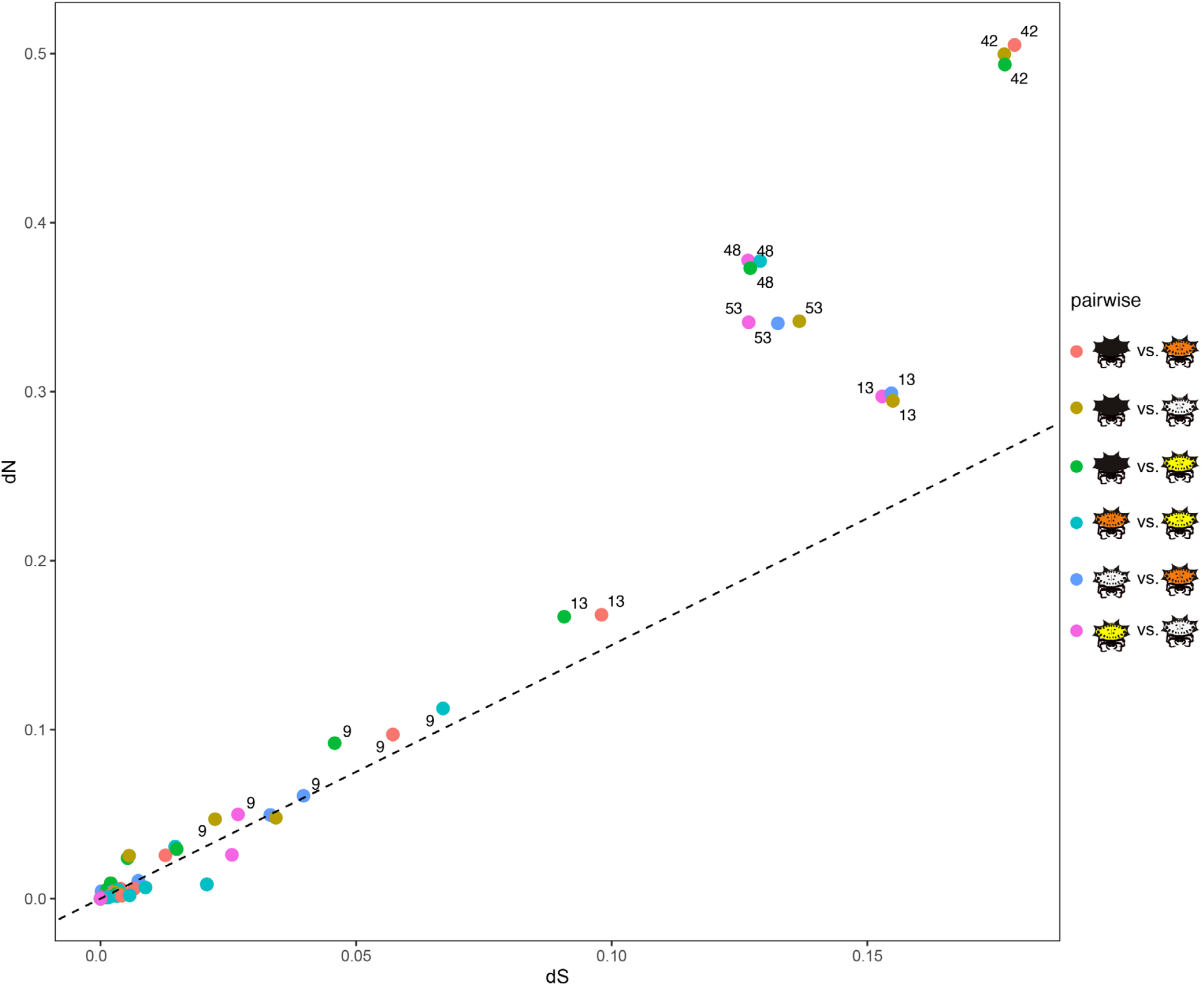
Plot of dN vs. dS values from pairwise analysis for pigment genes in Table 3. Numbers correspond to codes assigned in Table 3 and are shown exclusively for genes with significant signatures of positive selection.

When we evaluated for signatures of positive selection, we found five genes with significant *ω* > 1, and some showed color specific selection patterns (Figure 5 and Tables 3, S6). *BarH2* had *ω* > 1 in all pairwise comparisons, while the carotenoid gene *Cytochrome P450 18a1* showed significant signatures of positive selection in all but the yellow‐orange comparison. The melanin gene *Prophenoloxidase* 1 (PPO) also had *ω* > 1 exclusively in comparisons that involved the white morph, while the gene *Glut1*, likely involved in inhibiting melanization, showed positive selection only in the pairwise comparisons that included the black morph. Finally, the serine threonine *Ire1* had *ω* > 1 only in comparisons involving the yellow morph.

## 4 Discussion

The study of the genetic basis of animal coloration has usually been restricted to a few taxa and rarely addressed in spiders despite the fact they are among the most diverse animals on earth. However, high‐throughput sequencing technologies now allow us to address this question in more species and traits, which is crucial for understanding the evolution of animal coloration and its significance in nature. In this study, we generated a comprehensive transcriptome assembly for the spider *G. cancriformis* and identified candidate genes associated with abdominal pigmentation. Our results suggest that genes in the carotenoids, melanin, ommochromes, and pteridine pathways play a role in producing this female restricted phenotype, and the signatures of selection detected on some of these genes suggest an adaptive role for coloration likely via aposematism (as indicated by the upregulation of venom glands genes in colorful morphs). We also present the first assembled transcriptome of the color polymorphic spider *G. cancriformis*, which constitutes an improved reference with a higher N50, a longer total length, and fewer predicted genes compared to previous assemblies for the genus (Zhao et al. 2014). These assembly statistics are much more consistent with estimates from multiple spider species (Sanggaard et al. 2014; Yu et al. 2024; Fan et al. 2021) and may be the result of sequencing more than one individual and implementing a more stringent contig length threshold (> 2000 bp) than previous transcriptomic studies in *Gasteracantha*.

### 4.1 Genes Associated With Pigmentation

The presence of any coloration (yellow, orange, or black) seems to rely on the expression of ommochromes and pteridines. As such, ommochrome genes such as *loco, white*, and *hayan*, and pteridine genes such as *punch* and *NFI* were always overexpressed in pigmented phenotypes (Table 3). The role of these genes is related to both biosynthesis and transport of pigments, and thus consistent with our results. The association of *punch, NFI*, and *white* with ommochromes and pteridines in spiders has already been reported (Croucher et al. 2013). In contrast, this is the first study that associates *hayan* and *loco* with coloration in spiders, although they have been implicated in pigmentation in *D. melanogaster* , with their function not yet understood (Dembeck et al. 2015; Nam et al. 2012).

### 4.2 Yellow and Orange

The presence of yellow and orange coloration (but not black) had a genetic signature characterized by carotenoid, ommochromes, and pteridine related genes. Carotenoid genes like *cytochrome P450 18a1*, *astacin*, and *vitellogenin* were highly expressed in association with orange and yellow, which agrees with reports in multiple animal taxa where the production and oxidation of carotenoids, particularly astaxanthin, requires the cytochrome P450 complex and astacin metalloendopeptidases (Sirisidthi et al. 2015; Zhao et al. 2015; Choi and Koo 2005; Xing et al. 2017; Mundy et al. 2016; Weaver et al. 2020). Interestingly, *vitellogenin* is known to transport astaxanthin in the chum salmon *Oncorhynchus keta* (Ando et al. 1986), and it has been involved in promoting pigment deposition in red and chocolate strains of the shrimp *Symphysodon aequifasciatus* (Haque et al. 2023). Thus, our results suggest that the mechanisms underlying the biosynthesis and use of carotenoid in animals may be evolutionarily conserved and likely contribute to orange and yellow pigments in *Gasteracantha*. However, other studies have failed to detect carotenoids in some spider species, suggesting their role may be limited or context‐dependent (Croucher et al. 2013; Riou and Christidès 2010). To the best of our knowledge, these are the first candidate genes likely involved in carotenoid pigmentation reported in spiders.

We also observed that genes such as *cinnabar* and *pink* are highly expressed in association with yellow/orange coloration, and they have well‐characterized functions in ommochrome and pteridine synthesis (Reed and Nagy 2005) or transport (Futahashi and Osanai‐Futahashi 2021; Figon and Casas 2019; Sevrioukov et al. 1999; Myers et al. 2021). These genes have already been associated with ommochrome and pteridines in spiders (Croucher et al. 2013).

Interestingly, the absence of yellow and orange is genetically distinguishable by the overexpression of melanization proteases 1 and 2 (*MP1* and *MP2*), which are required for activating the tyrosinase and turning on the melanization cascade (Sugumaran and Barek 2016). Similarly, the presence of the yellow phenotype only or the orange phenotype only can be recognized based on the expression pattern of ommochrome, pteridine, and melanization genes. The absence of orange or yellow is related to genes involved in controlling melanization like *pinstripe*, which likely affects abdominal pigmentation in *D. melanogaster* through vesicle formation and transport (Dembeck et al. 2015; Orgogozo 2016), and others related to melanin control in a yet unknown manner, such as *buttonless* (Dembeck et al. 2015) or *Ire1* (Mitra and Ryoo 2021).

### 4.3 Black

Although melanin has long been thought to be absent in spiders, recent studies have documented the presence of eumelanin in several species (Hsiung et al. 2015, 2017; Politi et al. 2021). In agreement with this observation, we found 10 genes associated with the presence of the black phenotype in *G. cancriformis*, and 15 associated with its absence (Table 3). The known molecular function of these genes supports that melanin causes black coloration in the abdomen of this spider (Zhao et al. 2023). For example, we found the gene *Prophenoloxidase* 1 (PPO1) which encodes the inactive form of tyrosinase, a key copper‐dependent enzyme in the melanization cascade in arthropods (Charoensapsri et al. 2011). In the same way, we found the copper transporting ATPase—*ATP*, which is required for melanin production in flies likely by providing copper to the tyrosinase (Norgate et al. 2006). Also, the overexpression of the *bursicon* gene in the black morph, which is involved in the correct tanning in insects (Flaven‐Pouchon et al. 2020), agrees with the presence of the melanic phenotype. Interestingly, we observed overexpression of *Abdominal‐B* (Abd‐B), but downregulation of *ebony*. This agrees with the function of the Hox protein Abd‐B in activating the melanization gene *yellow* in the posterior segments of *D. melanogaster* only when *ebony* is downregulated (Jeong et al. 2006; Ferguson et al. 2011; Futahashi et al. 2008; Matsuoka and Monteiro 2018; Wittkopp et al. 2002). Additionally, the expression of the transcription factor *bric‐a‐brac* was associated with the absence of melanin, which agrees with its role as a modulator of orange and yellow pigmentation in butterflies (Loh et al. 2025; Hanly et al. 2023).

Other genes we found associated with the black morph have been implicated in pigmentation in arthropods, but their actual role in melanism is not yet understood. For example, the homeobox transcription factor *BarH2* is expressed in primary pigment cells in pupae of *D. melanogaster* (Kang et al. 2014; Higashijima et al. 1992), and the ommochrome synthesis gene *cardinal* catalyzes the conversion of 3‐hydroxykynurenin to xanthommatin (Futahashi and Osanai‐Futahashi 2021). Interestingly, the *Rho1‐*GTPase (Table 3) is somehow necessary for melanization in mosquitoes (Shiao et al. 2006), and the activation of Rho‐GTPases is controlled by Rho‐GTP exchange factors (GEFs) such as RhoGEF3, which was detected in our study as associated with the black morph (Kim et al. 2019; Hunter 2002). Similarly, the GTPase Rab32/RP1, encoded by the gene *lightoid* (Table 3), participates in the biogenesis or degradation of pigment granules in *D. melanogaster* and *Bombyx mori* (Liu et al. 2021).

Overall, the genes we found in the black morph indicate that the melanin synthesis pathway is active in black individuals of *G. cancriformis* , and along with previous melanin‐related genes already reported for spiders (Croucher et al. 2013; Zhao et al. 2023), we found new genes likely playing a role in melanism in these arthropods (Table 3).

### 4.4 White

The white morph of *G. cancriformis* showed upregulation of the *karneol* gene, which is involved in iridophore development (Bian et al. 2019; Krauss et al. 2014; Singh and Nüsslein‐Volhard 2015). Iridophores contain reflective guanine crystals (Singh and Nüsslein‐Volhard 2015; Hashimoto et al. 2021), which have been implicated in the production of white coloration in spiders, fishes, and squamates by scattering light (Hsiung et al. 2019). Similarly, chitin‐based microstructures possess optical properties that have been associated with structural color in multiple insects (Nakazato and Otaki 2023; Bai et al. 2022), and the *chitin synthase 2* gene, which is upregulated in the white morph, may play a role in the formation of such microstructures. Therefore, our results are consistent with the white color in *G. cancriformis* being the result of microstructures rather than pigments.

### 4.5 Signatures of Selection

The fact that we observed color‐related signatures of positive selection suggests that abdominal coloration in *G. cancriformis* could play an adaptive role still unknown. First, aposematism might advertise toxicity via color phenotype to avoid predation. Our observation that six genes with known expression in venom glands in animals (Barua and Mikheyev 2021; Pan et al. 2023; Ullah and Masood 2020; Rami et al. 2024; Chaim et al. 2006; Gu et al. 2022) are overexpressed in the yellow and orange individuals of *G. cancriformis* (Table 4) is indicative of these colorful morphs actively synthesizing more venom than the nonconspicuous phenotype, likely serving a defensive function against predators (Lüddecke et al. 2022; Ximenes and Gawryszewski 2020). In fact, previous studies in *Gasteracantha* found that brightly colored morphs are less frequently attacked by predators than their black and white counterparts (Gawryszewski and Motta 2012; Moya‐Laraño et al. 2013). However, a direct relationship between conspicuousness and aposematism in *G. cancriformis* requires experimental testing, especially because we also observed higher expression of toxin related genes in the black phenotype (which contradicts the conspicuousness hypothesis). Second, the overexpression of venom related genes in the yellow and orange individuals (Table 4) may indicate that these morphs attract more prey, thereby requiring greater venom production for prey immobilization and digestion. However, experimental evidence in *G. cancriformis* has shown that body coloration is not as relevant for prey attraction, and in fact, black spiders are more effective at attracting prey than conspicuous morphs (Salgado‐Roa et al. 2023; Gawryszewski and Motta 2012). Third, abdominal color polymorphism in *G. cancriformis* may be due to microhabitat segregation, where each morph occupies a distinct ecological niche that imposes a particular selection regime. Evidence supporting this scenario comes from the Christmas spider ( *Austracantha minax* ), where distinct color morphs occupy distinct climatic niches (Salgado‐Roa, Stuart‐Fox, and Medina 2024), and from *Timema* stick insects, where disruptive selection associated with shifts in color in the host plant maintains color polymorphism (Villoutreix et al. 2023). Fourth, disassortative mating coupled with frequency dependent selection may underlie color polymorphism in *G. cancriformis* . Although these mechanisms combined promote color polymorphism in the butterfly *Heliconius numata* (Chouteau et al. 2017, 2016), its role in polymorphic spiders needs experimental testing. Fifth, sexual selection could also explain color variation in these spiders if males choose mates based on visual cues. However, web‐building spiders have limited color vision and rely on chemical and vibrational signals for mate attraction (Yamashita 1985; Tiedemann et al. 1986). Consequently, color polymorphism in web‐building spiders may not be due to intrasexual interactions. Sixth, differential conspicuousness among *G. cancriformis* morphs may be associated with foraging success via flower mimicry (i.e., the “prey attraction hypothesis”, Ximenes and Gawryszewski 2019; Tso et al. 2006; Hauber 2002). However, recent studies have shown that, the position of the spider on the web is more important than coloration for prey capture in *G. cancriformis* (Salgado‐Roa et al. 2023).

Our sampling in a single polymorphic population allowed us to compare gene expression among color morphs and identify melanin related genes highly expressed in the black morph, carotenoid and ommochrome genes expressed in the yellow and orange morphs, and structural related genes in the white morph. To our understanding, this is one of the few studies that addresses this question in color polymorphic spiders, a group of animals not included in the most up to date catalog of color loci (Martin and Orgogozo 2013). More studies are needed to identify the master input–output genes (Stern and Orgogozo 2009) that trigger coloration development in arachnids.

## Author Contributions

**Paula Torres‐Quintero:** formal analysis (lead), methodology (equal), writing – original draft (equal). **Carolina Pardo‐Díaz:** conceptualization (equal), funding acquisition (equal), investigation (equal), project administration (equal), resources (equal), supervision (equal), validation (equal), writing – original draft (equal). **Fabian Salgado‐Roa:** formal analysis (supporting), investigation (supporting), resources (supporting), writing – review and editing (supporting). **Camilo Salazar:** conceptualization (equal), formal analysis (equal), funding acquisition (equal), investigation (equal), methodology (equal), project administration (equal), resources (equal), supervision (equal), validation (equal), writing – original draft (equal).

## Funding

This work was supported by the Universidad del Rosario (10.13039/501100008793).

## Conflicts of Interest

The authors declare no conflicts of interest.

## Supporting information


**Figure S1:** Distribution of transcripts by length (bp).
**Figure S2:** Functional annotation and classification of unigenes identified from the transcriptome of *G. cancriformis* . (A) KEGG annotation results are shown both as individual functions (with the number of genes mapping to each function at the side of the bar) and as KEGG metabolic pathways (five categories that are color coded and with the *x*‐axis indicating the percentage of genes annotated to a given category). (B) GO term annotations with Blast2GO classifying genes into individual functions (with the number of genes mapping to each function in the *y*‐axis) and into three main functional categories (color coded).

**Tables S1–S6:** ece373315‐sup‐0002‐Tables.xlsx.

## Data Availability

All raw sequencing data is available through NCBI Short Read Archive (ENA) database (accession number PRJEB94359). The code used to analyze the data is available in: https://paula‐torres.github.io/Genetic‐basis‐color‐Gasteracantha/.
