# Supplementary material for: The Genetic Basis of Color Polymorphism in the Orb‐Web Spider Gasteracantha cancriformis
Source: Ecol Evol. 2026 Mar 23;16(3):e73315. doi: 10.1002/ece3.73315 (PMC13093652; doi:10.1002/ece3.73315)
Supplement: Supplementary file 1 — Figure S1: Distribution of transcripts by length (bp). Figure S2: Functional annotation and classification of unigenes identified from the transcriptome of G. cancriformis . (A) KEGG annotation results are shown both as individual functions (with the number of genes mapping to each function at the side of the bar) and as KEGG metabolic pathways (five categories that are color coded and with the x‐axis indicating the percentage of genes annotated to a given category). (B) GO term annotations with Blast2GO classifying genes into individual functions (with the number of genes mapping to each function in the y‐axis) and into three main functional categories (color coded). [file ECE3-16-e73315-s001.docx]

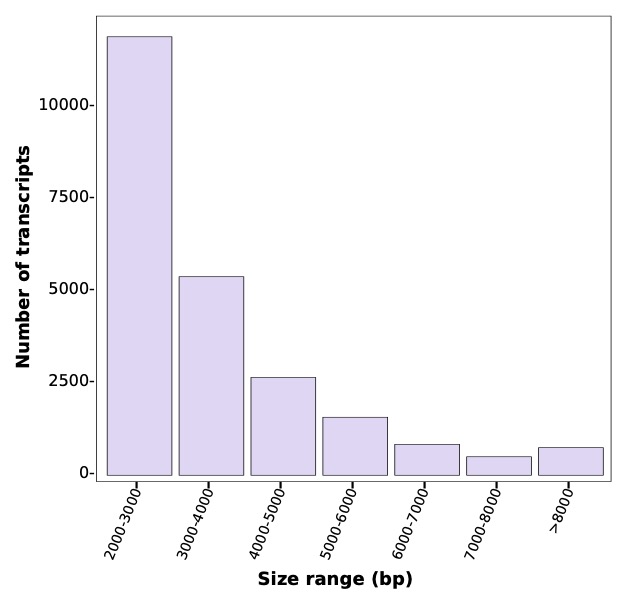


**Figure S1.** Distribution of transcripts by length (bp)


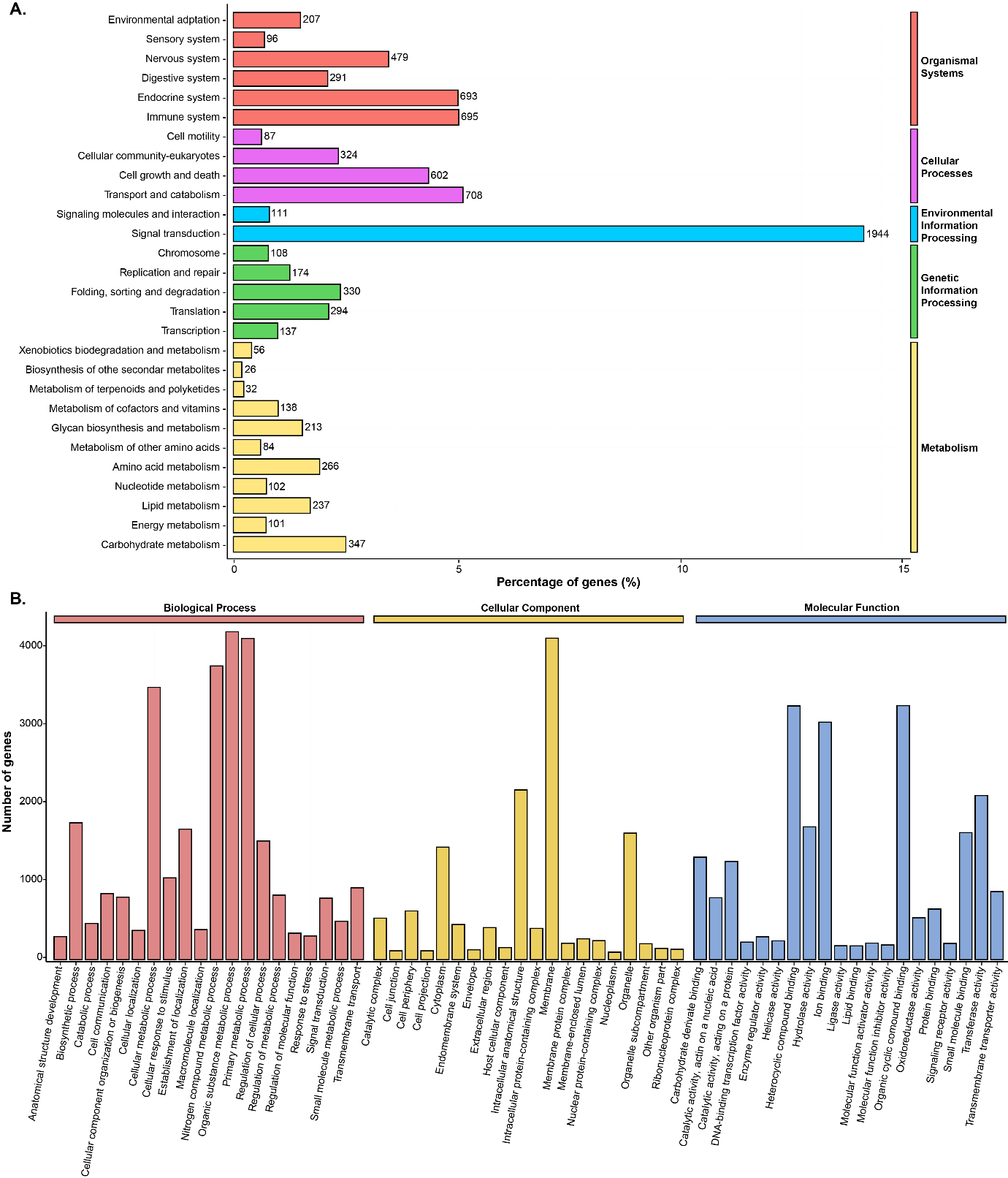


**Figure S2.** Functional annotation and classification of unigenes identified from the transcriptome of *G. cancriformis*. **A.** KEGG annotation results are shown both as individual functions (with the number of genes mapping to each function at the side of the bar) and as KEGG metabolic pathways (five categories that are color coded and with the x-axis indicating the percentage of genes annotated to a given category). **B.** GO term annotations with Blast2GO classifying genes into individual functions (with the number of genes mapping to each function in the Y axis) and into three main functional categories (color coded).
